# Role of acute alcohol‐induced acetylation in tauopathy

**DOI:** 10.1002/alz70855_103562

**Published:** 2025-12-24

**Authors:** Usman Sabir, Takhar Kasumov, Bovinari Alistair Csubak, Sameer Parashar, Andrea Arias Alvarado, Serguei Ilchenko, Christine M. Dengler‐Crish

**Affiliations:** ^1^ Neomed University rootstown, Rootstown, OH, USA

## Abstract

**Background:**

Alzheimer's disease (AD) poses a significant concern for aging populations, with evidence suggesting alcohol intake may accelerate brain aging and increase AD risk. Normal aging involves altered proteostasis and cognitive decline, regulated by ε‐lysine acetylation. In AD, tau and histone acetylation are disrupted, but alcohol's impact on acetylation‐dependent tauopathy remains unexplored.

**Method:**

This study examined acetylation in age‐dependent tauopathy across presymptomatic (3–5 months), progressing (11–14 months), and advanced (>16 months) disease stages in htau mice using immunoassays. The effects of time‐ and dose‐dependent acute alcohol exposure on tauopathy markers were assessed, with EtOH‐d6‐ and ²H₂O‐labeled mass spectrometry tracing alcohol's impact on cortical acetylation and tau turnover, respectively

**Result:**

In female htau mice, cortical acetylated tau (TauK174ac) increased with age and disease progression, correlating with total tau accumulation. In males, phosphorylated tau (TauS202p) increased without changes in Tau174ac, highlighting distinct sex‐dependent roles of acetylation and phosphorylation. A single EtOH dose (5 mg/g BW) transiently elevated Tau174ac (peaking at 2 hours) and progressively increased TauS202p and total tau in 9‐month‐old htau mice (*p* <0.05). Higher EtOH doses (3–5 mg/g BW) promoted acetylation‐dependent tau accumulation, while acute EtOH exposure elevated H4K16 and H3K9 acetylation across all doses, accompanied by increased CBP and MOF1 expression, with no changes in Sirt1, Sirt6, or other KATs and KDACs involved in tau or histone acetylation.

Mass spectrometry revealed that EtOH‐d6‐derived acetate contributed to histone acetylation but not tau acetylation. A ²H₂O‐based turnover study showed increased turnover rates for multiple proteins, but synuclein and tau exhibited reduced turnover in tauopathy mice compared to wild‐type controls. Additionally, tau proteoforms containing the K174 acetylation site (residues 171–194) had longer half‐lives than non‐acetylated forms, suggesting alcohol may indirectly exacerbate tauopathy by affecting tau turnover.

**Conclusion:**

These findings indicate that alcohol‐induced disruptions in brain acetylation may accelerate cognitive decline through epigenetic changes and impaired tau turnover. Females may be more susceptible to alcohol‐accelerated tauopathy due to elevated tau acetylation, potentially impairing protein degradation. Future studies will explore the effects of chronic alcohol consumption on tauopathy and whether alcohol accelerates tauopathy via histone acetylation‐mediated transcriptional changes in tau degradation pathways.

